# PRE- AND POSTOPERATIVE IN BARIATRIC SURGERY: SOME BIOCHEMICAL CHANGES

**DOI:** 10.1590/0102-6720201600S10017

**Published:** 2016

**Authors:** Amanda Kaseker TEDESCO, Rafaela BIAZOTTO, Telma Souza e Silva GEBARA, Maria Paula Carlini CAMBI, Giorgio Alfredo Pedroso BARETTA

**Affiliations:** Clínica Baretta, Curitiba, PR, Brazil

**Keywords:** Bariatric surgery, Dyslipidemia, Vitamin B12, Homocysteine, C reactive protein

## Abstract

**Background:**

The bariatric surgery may cause some nutritional deficiencies.

**Aim:**

To compare the serum levels of biochemical markers, in iimmediate post-surgical patients who were submitted to bariatric surgery.

**Methods:**

Non-concurrent prospective cross-sectional study. The analysis investigated data in medical charts of pre-surgical and immediate post-surgical patients who were submitted to bariatric surgery, focusing total cholesterol, HDL cholesterol, LDL cholesterol, triglycerides, C reactive protein, vitamin B_12_ levels, folic acid, homocysteine values, iron and serum calcium at the referred period.

**Results:**

Twenty-nine patients of both genders were evaluated. It was observed weight loss from 108.53 kg to 78.69 kg after the procedure. The variable LDL-c had a significant difference, decreasing approximately 30.3 mg/dl after the surgery. The vitamin B_12_ serum average levels went from 341.9 pg/ml to 667.2 pg/ml. The triglycerides values were in a range of 129.6 mg/dl-173.3 mg/dl, and 81.9 mg/dl-105.3 mg/dl at the pre- and postoperative respectively. CRP levels fall demonstrated reduction of inflammatory activity. The variable homocysteine was tested in a paired manner and it did not show a significant changing before or after, although it showed a strong correlation with LDL cholesterol.

**Conclusion:**

Eligible patients to bariatric surgery frequently present pre-nutritional deficiencies, having increased post-surgical risks when they don´t follow an appropriate nutritional follow-up.

## RESUMO

**Racional:**

A cirurgia bariátrica pode causar deficiências nutricionais.

**Objetivo:**

Comparar os níveis séricos bioquímicos de pacientes submetidos à cirurgia bariátrica no pré e pós-operatório precoce.

**Métodos:**

Estudo transversal, retrospectivo não concorrente. A análise considerou a investigação de prontuários de pacientes submetidos à gastroplastia no período pré-operatório e pós-operatório precoce, analisando resultado bioquímicos de colesterol total, HDL colesterol, LDL colesterol, triglicérides, proteína C reativa, dosagens de vitamina B_12_, ácido fólico, valores de homocisteína, ferro e cálcio séricos, no referido período.

**Resultados:**

Compuseram a amostra 29 pacientes de ambos os sexos. Houve redução de peso após o procedimento cirúrgico com média de 108,53 kg para 78,69 kg. A variável LDL-c apresentou diferença significativa com diminuição de aproximadamente 30,3 mg/dl após a gastroplastia. Com relação à média de níveis séricos de vitamina B_12_ ela passou de 341,9 pg/ml para 667,2 pg/ml. Os valores de triglicérides encontravam-se na faixa de 129,6 mg/dl-173,3 mg/dl, e 81,9 mg/dl-105,3 mg/dl no pré e pós-cirúrgico, respectivamente. Foi evidenciada redução da atividade inflamatória verificada mediante queda dos níveis de PCR. A variável homocisteína foi avaliada de maneira pareada e não apresentou mudança significativa no antes e depois, havendo, contudo, forte correlação com o LDL-colesterol.

**Conclusão:**

Pacientes candidatos à cirurgia bariátrica frequentemente apresentam deficiências nutricionais anteriores ao procedimento com riscos aumentados no período pós-cirúrgico quando não aderem ao acompanhamento nutricional adequado.

## INTRODUCTION

Bariatric surgery is indicated for obese patients with a body mass index (BMI) greater than 40 kg/m², or even between 35-39.9 kg/m^2^ with comorbidities. The indication has grown significantly. In Brazil it was performed 60,000 operations in 2010, representing an increase of 33% the previous year, and 275% compared to 2003^12^ ^,^ ^24^.

Several diseases are associated with obesity and dyslipidemia are among the most frequent ones. Both conditions have considerable potential of morbidity and mortality in relation to altered levels of serum lipids, as well as the increased risk of atherogenesis, systemic arterial hypertension, insulin resistance and coagulation disorders. The prevalence of dyslipidemia is variable among patients with morbid obesity (19.0-82.9%). Some studies consider dyslipidemia when only one of the serum lipid levels are alterated: total cholesterol, high density lipoprotein (HDL cholesterol), low density lipoproteins (LDL-cholesterol) and triglycerides, also known as primary dyslipidemia; while others, only when there is mixed dyslipidemia (hypercholesterolemia associated with hypertriglyceridemia)^15^ ^,^ ^19^ ^,^ ^27^.

The objective of this study was to compare serum biochemical levels in pre- and postoperative of bariatric surgery.

## METHODS

This research was authorized by the Committee of Ethics and Research of Positivo University, under n^o^. 1.073.415, meeting the requirements of resolution 466/2012 from the National Commission on Ethics in Research of CONEP^4^.

A cross-sectional, retrospective and non-concurrent study was held in a private clinic for patients monitoring pre- and postoperative (PO) of bariatric surgery at the Clinic Baretta, Curitiba, PR, Brazil. The analysis obtained was through investigation of the records of patients undergoing gastroplasty from January to September 2015, in the pre- and postoperative period (nutritional monitoring within the first postoperative year) and data collection was held between the months of July to September 2015. For the sample, 29 charts of patients of both genders were selected, with ages between 18-70 years old, which underwent Roux-en-Y gastric bypass for obesity correction. The analysis considered the biochemical assessment of total cholesterol (TC), high density lipoprotein (HDL-c), low density lipoprotein (LDL-cl), triglycerides (TG), C-reactive protein (CRP), serum dosages of vitamin B_12_, folic acid, serum homocysteine, iron and calcium, in two moments: preoperative and the first nutritional monitoring consultation in postoperative. Were considered: gender, age, weight, height, and current BMI in the same monitoring periods.

For the values of serum lipid profile, a dosage of TC equal to 200 mg/dl was used, having as preventive goal those distributed in LDL-cholesterol <160 mg/dl, triglycerides <150 mg/dl and HDL-cholesterol>40 mg/dl, in men, and >50 mg/dl in women, classifying them in desirable, high and low, and blood glucose of 70-100 mg/dl^24^

For dosages of vitamin B_12_ and folate, the following reference values were given: 200 to 900 pg/ml and 9,8 and 16,2 nmol/l respectively^10^ ^,^ ^29^. For homocysteine values (Hcy), the same adopted for the population under 70 years was established as reference: 6-12 µmol/l for women and 8-14 µmol/l for men^29^.The dosage adopted for serum calcium (serum or plasma) was 8,8-11,0 mg/dl. The values adopted for serum iron according to gender were, for men, 59-158 mcg/dl and, for women, 37-145 mcg/dl^29^. For C-reactive protein the values considered were <1.0 mg/l or <0.1 mg/dl for low risk^18^.

Data collection considered biochemical results coming from more than one clinical analysis laboratory, and were excluded the medical charts incompletely fulfilled.

### Statistical analysis

Were correlated the homocysteine serum levels with the risk of cardiovascular diseases in gastrectomized patients, seeking to identify possible nutritional inadequacies relating them to the levels of vitamin B_12_ and folic acid in this population, comparing the C-reactive protein values in the pre- and postoperative. Statistical analysis was performed using R-tool (R Development Core Team 2015, version 3.2.2). For the descriptive analysis, the package cvforecastLopes^14^ was used.

## RESULTS

The sampling was composed by 29 patients of both genders, 26 women and three men. The Table 1 shows that the average age of the population was 35.86 years old (18-67). Faced with the analysis of the average weight variable, there was a significant reduction after surgery of 108.53 kg to 78.69 kg with standard deviation of 16.03 kg and 15.07 kg, respectively. Consequently, the average BMI presented reduction from 40.06 kg/m^2^ to 28.75 kg/m^2^, causing the rating decrease from morbidly obese to overweight (SD=5.01 and 4.51), representing a decrease of 28.23% in the overall average weight. By subtracting the standard deviation of the average in the postoperative period, the BMI reached 24.24 kg/m^2^, expressing as final results the following classifications: 27% of patients remained in the obesity range, 51.7% with overweight and 20.7% in the range of eutrophy. 

**TABLE 1:** Age and comparison among values of weight, height and BMI in pre- and postoperative periods

Variable	Average (BI 95%)			Median		Standard deviation
	Before	After	Before	After	Before	After
Age*	35.86(30.7; 40.4)	35.9(30.7; 40.6)	31	31	13.57	13.57
Weight (kg)	108.5(103.2; 114.3)	78.7(72.9; 83.6)	107	74.3	16.03	15.07
Heigth (m)	164.5(161.4; 167.4)	164.5(161.5; 167)	163	163	8.08	8.08
BMI (kg/m²)	40.1(38.2; 41.5)	28.7(27.1; 30.1)	39.4	28	5.01	4.51

B=before; A=after; *years old

The average value of total cholesterol in the studied group had a decrease of 19%, going from 200.78 mg/dl to 162.63 mg/dl in average; however, when assessing the values of HDL-c, no significant alterations were observed. The LDL-c variable presented a significant difference with decrease of approximately 30.3 mg/dl after the gastroplasty.

For the assessment of the lipid profile of patients that underwent the gastric bypass in the pre- and postoperative periods, the covariates TC (Wilcoxon, p=0.0000), LDL (test t, p=0.0001) and HDL (Wilcoxon, p=0.9184) were applied.

As to the LDL-c before surgery, 44.8% of patients were presented within the ranges of 86.3-121 mg/dl; from these, 10.3% raised to the range 121-155 mg/dl and 17.2% lowered to the range 51.7-86.3 mg/dl (Table 2).

**TABLE 2:**
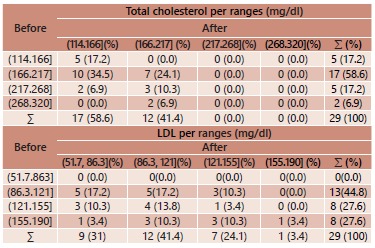
Ranges of TC, LDL-c, before and after the procedure

B=before; A=after; ∑=sum

By seeing the margins in Table 2, it is possible to conclude that, before the operation, 58.6% of patients had total cholesterol in the range of 166-217 mg/dl and, among these, 34.5% decreased to the range of 114-166 mg/dl in postoperative period. From 6.9% in the higher range, from 268-320 mg/dl all of them decreased to 166-217 mg/dl.

Indicators of vitamin B_12_, total triglycerides, folic acid, serum glucose, serum iron and calcium, and C-reactive protein (CRP) also presented some change (Table 3).

**TABLE 3:** Comparison among values of vitamin B12, TG, homocysteine, HDL-c, LDL-c, folic acid, CRP, glucose, serum iron and calcium in the pre- and postoperative periods

Variable	Average (BI 95%)			Median		Standard deviation
	B	A	B	A	B	A
Vit. B12	341.9 (295.1; 383.3)	667.2 (499.5; 807)	300	607	125.5	431.5
TG	149.8 (129.6; 173.3)	94.4 (81.9; 105.3)	149	92	58.04	31.16
Homocysteine	8.99 (8.2; 9.8)	8.6 (7.8; 9.4)	9.1	8.3	2.21	2.27
HDL	44.69 (39.9;48.6)	45.3 (40.5;49.8)	43	41.0	11.77	13.45
LDL	131.9 (120.4;143.1)	101.6 (89.7;113.7)	131	95.4	33.02	32.19
Folic acid	9.3 (7.6; 10.9)	12.5 (10.7; 14.6)	8.40	13.9	4.63	5.65
CRP	8.4 (6.2; 10.5)	3.5 (2.4;4.5)	8.80	3.4	6.31	3.17
Glucose	90.3 (85.1; 94.9)	81.7 (78.8;84.7)	86	81	12.52	7.96
Fe	92.1 (80.7; 102.9)	76.9 (68.1;86.5)	84	82	31.25	25.41
Ca	8.8 (8.4; 9.4)	9.1 (8.9;9.3)	9.10	9.2	1.50	0.46

B=before; A=after

In this study in the preoperative period the average of serum levels of vitamin B_12_ was 341.9 pg/ml. Afterwards, 667.2 pg/ml was observed, which is considered normal level.

Triglyceride levels in preoperative period were in the range of 129.6-173.3 mg/dl, and in the postoperative had considerable reduction, 81.9-105.3 mg/dl.

The analysis of levels of folic acid was 7.6-10.9 nmol/l before the operation and 10.7-14.6 nmol/l after surgery, demonstrating none significant improvement.

Serum iron was in the range of 80.7-102.9 μg/dl and after the intervention dropped down to 68.1-86.5 μg/dl.

 The analysis of calcium had no significant change, staying in the range of 8.4-9.4 mg/dl and 8.9-9.3 mg/dl in the pre- and postoperative periods, respectively.

To assess the inflammatory process caused by obesity from the CRP levels, comparing the pre- and post-surgery periods, the variable was analyzed in both. In Table 4 it is possible to see CRP levels lower than 6.56 mg/dl were present in 41.4% before, and after they came to 34.5%. In the range 6.56-13.1 mg/dl, the levels were 44.8% before and came to 6.9%, evidencing that 37.9% of patients presented decrease in the levels of this protein.

**TABLE 4:** Distribution of pre- and post levels of CRP

Before	CRP per ranges (mg/dl)				
	After				
	(0.6,56] (%)	(6.56,13.1] (%)	(13.1,19.7] (%)	(19.7,26.3] (%)	∑ (%)
(0.6,56]	10 (34.5)	2 (6.9)	0 (0.0)	0 (0.0)	12 (41.4)
(6.56,13.1]	11 (37.9)	2 (6.9)	0 (0.0)	0 (0.0)	13 (44.8)
(13.1,19.7]	2 (6.9)	0 (0.0)	0 (0.0)	0 (0.0)	2 (6.9)
(19.7, 26.3]	0 (0.0)	2 (6.9)	0 (0.0)	0 (0.0)	2 (6.9)
∑	23 (79.3)	6 (20.7)	0 (0.0)	0 (0.0)	29 (100)

B=before; A=after; ∑=sum

**TABLE 5:** Homocysteine and LDL-c per range

Homocysteine (umol/l)	LDL-c (mg/dl)				
	(51.7,86.3] (%)	(86.3,121] (%)	(121,155] (%)	(155,190] (%)	∑ (%)
(4.03,6.53]	4 (13.8)	0 (0.0)	1 (3.4)	0 (0.0)	5 (17.2)
(6.53,9.02]	3 (10.3)	8 (27.6)	2 (6.9)	0 (0.0)	13 (44.8)
(9.02,11.5]	1 (3.4)	4 (13.8)	2 (6.9)	0 (0.0)	7 (24.1)
(11.5,14]	1 (3.4)	0(0.0)	2 (6.9)	1 (3.4)	4 (13.8)
∑	9 (31)	12 (41.4)	7 (24.1)	1 (3.4)	29 (100)

B=before; A=after; ∑=sum. Fisher's exact test. p=0.0292

In this study, the Hyc variable was tested in a paired manner and did not present a significant change before and after, due to the number of the sample; however, it showed a strong correlation with the LDL-c, justified by the fact that homocysteine accelerates the oxidation of LDL-c, further increasing the risk of cardiovascular disease.

## DISCUSSION

The weight loss following gastric bypass is accompanied by sharp improvement of all obesity-related comorbidities. With the weight loss an improvement in insulin resistance occurs, as well as reduction in adiposity and increased metabolic control^20^, thereby, it reduces the cardiovascular risk factors. Surgical intervention in just three months proved to be effective in improving the lipid profile and, consequently, major cardiovascular risk factors. This improvement observed by reduction of TC, LDL-c, TG was also observed in other studies. Asztalos *et al*.^2^ demonstrated improvement in concentrations of LDL-c and TG from the first postoperative month. Nassif *et al*.^17^ found reduction in TC, LDL-c and TG, after four months. However, Vila *et al.* ^31^ reported reduction of TG, only starting from the sixth month.

In the early postoperative period may occur some nutritional complications such as vomiting. diarrhea and dumping syndrome^5^.

The vitamin B_12_, as well as iron and folic acid, are essential for cell development and division, as well as for the production of red blood cells, genetic material and myelin. The nutritional deficit that entails such a situation can be explained by lack of food and adequate supplementation in the preoperative period. Their deficiencies can cause pernicious anemia. neurological symptoms and weakness^5^. 

The TG is formed from carbohydrates and stored in cells as caloric reserve, being used for energy in food deprivation periods. Their excess can cause inflammatory cytokines that are commonly increased in obesity. According to studies in the literature, low levels of TG show lower risk for cardiovascular disease^30^

The absorption of folic acid takes place preferably in the duodenum; however, it can also occur along the entire length of the small intestine, as a result of postoperative physiological adaptation. The vitamin B_12_ is required for the conversion of methyl tetrahydrofolic acid (inactive) into tetrahydrofolic acid (active). Therefore, deficiency of vitamin B_12_ may result in folic acid deficiency^3^. 

Oliveira et al. demonstrated that conventional therapies are ineffective when compared to the effectiveness that gastric bypass patients have in overweight and impaired glucose metabolism disorders. A possible explanation for the improvement of blood glucose would be the immediate and severe deprivation of nutrients that occurs after surgery. According to Carvalho *et al*.^5^ studied 47 obese patients that underwent Roux-en-Y gastric bypass; among them, 15 had diabetes and five glucose intolerance. One year after the surgery; the 20 patients had normal levels of fasting plasma glucose and glycosylated hemoglobin. Results show that there are benefits obtained in glycemic control with gastric bypass, even with non-exclusive indication regarding the glycemic status.

With respect to serum iron, the levels found are in agreement with the literature, since they are affected due to decrease in food intake supplies, decreased production of gastric acid, hampering digestion and also due to the modification of the duodenum and proximal jejunum, primary absorption sites, where most of the iron is absorbed. It is important that the serum iron levels be monitored regularly. Some studies suggest that vitamin C supplementation can help in iron deficiency for assisting in the absorption process^6^ ^,^ ^23^.

Regarding the mineral calcium, obese people may have lower levels even before the surgery, and possible explanations include the reduction of physical activity with less exposure to sunlight and body fat storage increase with reduced bioavailability. The calcium absorption occurs in the small intestine and, even in patients with normal calcium levels, after gastric bypass the levels are affected, thus having depletion of this ion with consequences in bone architecture^7^. According with the literature, the data found are related to the surgical technique, because Roux-en-Y gastric bypass has no great result compared to other techniques^16^.

Epidemiological studies have documented recently that mild elevations in concentrations of CRP in the acute stage, even within the reference range, may predict the onset of cardiovascular disease and diabetes^1^. Obese patients may have high levels of CRP due to increased production of interleukin-6 and tumor necrosis factor in adipocytes, regulating hepatic production of CRP and inducing a state of chronic low-grade inflammation. Some evidences suggest that after gastrectomy the serum levels of CRP decrease 65%, (in average) according to the weight loss^25^. The reduction of inflammatory activity observed by decreasing levels of CRP in this study corroborates the results demonstrated by the researcher, who observed a decrease in the amounts of CRP provided by the occurrence of weight loss in the research patients^8^.

Homocysteine is a toxic amino acid derived from the amino acid methionine sulphide found in proteins from meat, dairy products, eggs, fishes and other sources. The increase of its levels has been reported after bariatric surgery. Its plasma concentration is influenced both by nutritional factors as hereditary ones, as well as pathological conditions such as decreased renal function and hypothyroidism^28^. Some researchers emphasize the role of Hcy only as a marker of folic acid deficiency and vitamin B_12_; others point out that the increase in their levels causes oxidative stress, which promotes neurological and vascular damage at the central level^27^. High levels of Hcy may indicate not only low levels of folate, but also an independent risk factor for cardiovascular disease and/or oxidative stress. The vitamins B_6_, B_12_ and folic acid are responsible for maintaining the concentration of homocysteine normalized^22^.

It is important to detect nutritional deficiencies in preoperative period, even if laboratory tests show limitations. This avoids difficulties on treatment in the postoperative period and, also, to not attribute mistakenly the micronutrient deficiency to the surgery^26^.

The most common deficiencies include iron, folic acid and vitamin B_12_ deficiency, due to the limited intake of animal proteins, due to the reduction of gastric secretions that impair the cleavage of the vitamin from the protein and inappropriate secretion of intrinsic factor. After the surgery, monitoring the levels of these elements is necessary^21^.

Changes in eating habits in the postoperative phase are factors that contribute to the emergence of the deficiencies of vitamins and minerals. The reduced intake of iron-rich foods, combined with the physiological changes brought about by the surgery, are considered a risk factor for development of anemia^11^. 

According to Leiro et al.^12^ nutrient supplementation after bariatric surgery is needed in all surgical techniques. In 2013 the guidelines given by American Association of Endocrinologists, the Obesity Society and the American Society for Metabolic and Bariatric Surgery have been updated, including 74 recommendations for patients in pre- and postoperative periods of bariatric surgery. In the case of gastric bypass, should be given a particular importance with regard to the possible deficiencies of iron, calcium, vitamin D, vitamin B1, vitamin B12 and folate^3^.

Micronutrient deficits are the main changes that jeopardize the success of surgical procedures. Nutritional supplementation in the long term, although much emphasized, still represents an obstacle to the success of surgical treatment of obesity. The start of supplementation or reposition of iron in an isolated manner has been recommended right after the hospital discharge, or 48 hours after the surgery. The use of preventive supplementation should compose the care protocol for all patients undergoing (or undergone) bariatric surgery. The treatment of nutritional deficiencies should consider mega doses of micronutrients due to lower bioavailability arising out of the physiological changes caused by surgical techniques^3^.

For greater success after surgery it is necessary to, in the preoperative period, strengthen the patient's perception that weight loss is possible when the energy balance becomes negative. It is required to identify dietary mistakes and disorders, inform the significant changes that the patient will face, start adjustments in the individual's power to promote real expectations of weight loss, prepare the patient for new food and habits and check the patient's potential for success operation^15^. According to Endevelt *et al.* ^9^ the follow-up of a nutritionist in the postoperative period is important. What determines the weight loss after surgery are the factors involved, and must to be taken into account the monitoring of surgery in the long term as well, to prove the benefits of the procedure.^23^ The research has shown significant reduction of BMI and nutritional deficiencies in patients with at least two follow-ups after the surgery, as well as improvement in lipid metabolism and in reducing the risk of cardiovascular disease. 

## CONCLUSION

The bariatric surgery was effective for weight loss and improvement of lipid metabolism. There was lack of vitamin B_12_ and folic acid. The calcium remained within normal limits. The C-reactive protein decreased after the surgery, demonstrating decrease in inflammatory process.
